# Reactive Oxygen Species Initiate Defence Responses of Potato Photosystem II to Sap-Sucking Insect Feeding

**DOI:** 10.3390/insects13050409

**Published:** 2022-04-24

**Authors:** Ilektra Sperdouli, Stefanos S. Andreadis, Ioannis-Dimosthenis S. Adamakis, Julietta Moustaka, Eleni I. Koutsogeorgiou, Michael Moustakas

**Affiliations:** 1 Institute of Plant Breeding and Genetic Resources, Hellenic Agricultural Organization–Demeter (ELGO-Dimitra), 57001 Thermi, Greece; ilektras@bio.auth.gr (I.S.); ekoutsogeorgiou@gmail.com (E.I.K.); 2 Section of Botany, Department of Biology, National and Kapodistrian University of Athens, 15784 Athens, Greece; iadamaki@biol.uoa.gr; 3 Department of Botany, Aristotle University of Thessaloniki, 54124 Thessaloniki, Greece; moustaka@plen.ku.dk

**Keywords:** chlorophyll fluorescence imaging, *Halyomorpha halys*, *Solanum tuberosum*, biotic stress, photoprotection, herbivore insects, singlet oxygen, non-photochemical quenching, photosynthetic efficiency

## Abstract

**Simple Summary:**

Potato is one of the most universally cultivated horticultural crops and is vulnerable to a range of herbivorous insects. One of them is the brown marmorated stink bug, an invasive polyphagous sap-sucking agricultural insect pest that penetrates the phloem to sieve elements and removes sap via a specialized mouthpart, the stylet. By using the chlorophyll fluorescence imaging methodology, we examined potato photosystem II (PSII) photochemistry responses in the area of feeding on the whole leaf area. Highly increased reactive oxygen species (ROS) generation was observed as rapidly as 3 min after feeding to initiate defence responses and can be considered the primary plant defence response mechanism against herbivores. Our experimental results confirmed that chlorophyll fluorescence imaging methodology can detect spatial heterogeneity of PSII efficiency at the whole leaf surface and is a promising tool for investigating plant response mechanisms of sap-sucking insect herbivores. We suggest that PSII responses to insect feeding underlie ROS-dependent signalling. We conclude that the potato PSII response mechanism to sap-sucking insect herbivores is described by the induction of the defence response to reduce herbivory damage, instead of induction of tolerance, through a compensatory photosynthetic response mechanism that is observed after chewing insect feeding.

**Abstract:**

Potato, *Solanum tuberosum* L., one of the most commonly cultivated horticultural crops throughout the world, is susceptible to a variety of herbivory insects. In the present study, we evaluated the consequence of feeding by the sap-sucking insect *Halyomorpha halys* on potato leaf photosynthetic efficiency. By using chlorophyll fluorescence imaging methodology, we examined photosystem II (PSII) photochemistry in terms of feeding and at the whole leaf area. The role of reactive oxygen species (ROS) in potato’s defence response mechanism immediately after feeding was also assessed. Even 3 min after feeding, increased ROS generation was observed to diffuse through the leaf central vein, probably to act as a long-distance signalling molecule. The proportion of absorbed energy being used in photochemistry (Φ*_PSII_*) at the whole leaf level, after 20 min of feeding, was reduced by 8% compared to before feeding due to the decreased number of open PSII reaction centres (q*p*). After 90 min of feeding, Φ*_PSII_* decreased by 46% at the whole leaf level. Meanwhile, at the feeding zones, which were located mainly in the proximity of the leaf midrib, Φ*_PSII_* was lower than 85%, with a concurrent increase in singlet-excited oxygen (^1^O_2_) generation, which is considered to be harmful. However, the photoprotective mechanism (Φ*_NPQ_*), which was highly induced 90 min after feeding, was efficient to compensate for the decrease in the quantum yield of PSII photochemistry (Φ*_PSII_*). Therefore, the quantum yield of non-regulated energy loss in PSII (Φ*_NO_*), which represents ^1^O_2_ generation, remained unaffected at the whole leaf level. We suggest that the potato PSII response to sap-sucking insect feeding underlies the ROS-dependent signalling that occurs immediately and initiates a photoprotective PSII defence response to reduce herbivory damage. A controlled ROS burst can be considered the primary plant defence response mechanism to herbivores.

## 1. Introduction

Worldwide crop production damages caused by foliage-feeding insects every year are estimated to be 5–30%, and the damage can be as high as 50% in the absence of insecticide application [1]. Two main herbivorous plant-dwelling insects can be recognized: those that chew and those that suck sap [2]. Chewing insects such as Colorado potato beetles (*Leptinotarsa decemlineata*) [2], potato pinworm (*Tuta absoluta*) [3], or tomato beet armyworm (*Spodoptera exigua)* [4], and grasshopper species induce severe plant tissue damage, while the sap-sucking insects instead cause indirect tissue destruction [2].

The plant vascular system is built up by the phloem and xylem tissues. The phloem is composed of sieve elements, companion cells, and phloem parenchyma cells [5]. The sieve elements are a pressure system that contains the phloem sap in which carbohydrates, proteins, and amino acids are diluted, thus, making it a favourite target of phloem-feeding insects [6]. The sap-sucking insects locate phloem sieve elements, penetrate cell walls, and extract sap via a specialized mouthpart, the stylet [2]. During stylet penetration, two types of saliva are secreted: the sheath saliva that gels soon after it is secreted (also called gelling saliva), and the watery saliva, which does not gel and is secreted into the sieve element [7]. The phloem sap-feeders are capable of retaining the penetrated sieve element and preventing or reversing the sieve element sealing response that stops the flow of sap to the pierced sieve element [7]. To protect the content and integrity of the sieve tubes against sap-sucking insects, plants retain miscellaneous chemical and physical defence mechanisms to impede the flow of sap by accumulating callose and phloem proteins [6,7], while herbivores, in response, have evolved adaptations to overcome these mechanisms [4,8]. In many circumstances, plant defence mechanisms encompass toxic or unpleasant compounds [7].

Plants emit volatile organic compounds (VOCs), and the profiles of emissions depend on the plant species [9]. Thus, when plants emit a particular compound, it is clearly detectable to particular insects that utilize the plant [9,10]. The aim of the released VOC by plants in response to an insect attack includes directly preventing herbivores, indirectly attracting natural enemies of attackers, and priming defences of intact organs on the same plant [11,12,13]. Miscellaneous molecular mechanisms regulate the interactions between plants and herbivore insects and the concomitant compensatory processes in the plants [4,14,15]. Most chewing insects feed on leaves or shoots, while others feed exclusively on roots or seeds [2]. Heavy infestations of sap-sucking insects cause long-lasting shortages of photosynthates and lead to a severe reduction in plant growth [2,16]. In order to understand the extensive range of plant responses to insects, an evaluation of how feeding affects the plant’s physiology [17], especially photosynthesis, is needed [16]. Sap-sucking insects impose a more severe negative impact on plant physiology than the chewing insects do, mostly due to the lower capabilities of plants to counterbalance sap-feeder damage in terms of growth and photosynthesis [18].

Together with several other biotic stresses, insect herbivory is known to alter photosynthesis activity (mostly negatively [3]), although compensatory responses are not exceptional [4]. Photosynthesis is allocated into two distinct parts, the electron transfer process and the carbon dioxide fixation. The absorbed light energy by the light-harvesting chlorophyll-protein complexes (LHCII) of photosystem II (PSII) is transferred into the reaction centre (RC), where water oxidation is taking place. Electrons from the water oxidation are transmitted from plastoquinone, via cytochrome *b_6_f* complex (Cyt *b_6_f*), to plastocyanin (PC), reaching through photosystem I (PSI), to ferredoxin (Fd), to reduce NADP^+^ and form NADPH [19]. Meanwhile, the proton gradient that is established across the thylakoid membranes is employed for ATP synthesis. NADPH and ATP are then used by the plant to maintain growth and development. Under almost all kinds of stresses, the light energy absorbed by PSII and PSI overdoes the amount that can be used for photochemistry, resulting in an increased formation of reactive oxygen species (ROS), such as singlet oxygen (^1^O_2_), superoxide anion radical (O_2_**^•−^**), and hydrogen peroxide (H_2_O_2_) [20,21,22,23,24,25,26]. In order to avoid photoinhibition, mainly of PSII [27,28,29,30,31,32,33], PSI photoinhibition is, to a minor level, likely to occur [33,34]. Plants have developed various mechanisms to control the absorbed light energy [35,36]. Non-photochemical quenching (NPQ) is one of the fundamental mechanisms that prevent photoinhibition in plants [35,36,37,38].

Plant cells are continuously producing ROS at basal levels, which are unable to cause harm, as they are being scavenged by different antioxidant mechanisms [23,39,40,41,42,43]. ROS–antioxidant interactions provide fundamental information for the redox state that impacts gene expression associated with abiotic and biotic stress responses regulating the induction of photosynthetic acclimation or cell death to maximize defences [39,44,45,46,47]. Despite their damaging activity, ROSs are defined as second messengers in a diversity of developmental and cellular processes [40,41], including biotic stress defences [3,4,42]. The role of chloroplast antioxidants, which often have overlying or interconnecting functions, is not to completely remove O_2_^•−^, H_2_O_2_, and ^1^O_2_. However, to accomplish a proper equilibrium between creation and removal so that to pair with the process of photosynthesis and warranty an effective spread of signals to the nucleus [39,44,45,46,47].

Biotic stresses primarily diminish the photosynthetic efficiency of plants having negative effects on photosystem II photochemistry and electron transport mechanisms [1,3]. A better understanding of plant primary photochemistry under biotic stress can help in managing the stress. Chlorophyll fluorescence imaging offers a quick, high-resolution, non-destructive technique to observe the spatial variation in PSII activity following herbivore harm [3,4,48,49,50,51].

Potato, *Solanum tuberosum* L., is one of the most commonly cultivated horticultural crops throughout the world, being the world’s fourth-highest important food crop, after maize, wheat, and rice, and is susceptible to a variety of herbivory insects [3]. One of these is the brown marmorated stink bug, *Halyomorpha halys* Stål (Hemiptera: Pentatomidae), an invasive polyphagous agricultural insect with the potential to become a serious economic threat, infesting more than 100 species besides potato, including many crops, where it causes severe economic losses [17]. *Halyomorpha halys* is a sap-sucking insect that penetrates the phloem sieve elements and removes the sap via a specialized mouthpart, the stylet (Figure S1). Heavy infestations of sapsucking insects cause long-lasting shortages of photosynthates and lead to a severe decrease in plant growth [18].

In the present study, we evaluated the outcome of short feeding periods of *H. halys* on potato leaf photosynthetic efficiency and examined if photosystem II (PSII) photochemistry of the whole leaf is influenced differentially by the feeding zone. We also assessed if any defence response mechanism is activated and whether ROSs are implicated in the response mechanism and are induced immediately after feeding to act as second messengers and activate the potato’s resistance or tolerance mechanism.

## 2. Materials and Methods

### 2.1. Plant Material and Growth Conditions

*Solanum tuberosum* L. cv Spunta (potato) plants were grown-up in pots containing peat moss (Terrahum) and perlite (Geoflor) (1:1 *v*/*v*), in an insect-proof greenhouse, under 22 ± 2 °C day temperature, 18 ± 2 °C night temperature, 68 ± 3% relative humidity, and natural light.

### 2.2. Insect Colony

*Halyomorpha halys* adults were obtained from a laboratory colony maintained at the Entomology Lab of the Institute of Plant Breeding and Genetic Resources (Thermi, Greece). This colony was established in the fall of 2019, originating from mixed-sex adults that were collected from households and fields in central Macedonia (Greece). Both adults and nymphs were placed in mesh cages (30 × 30 × 30 cm) with vinyl windows and zip closures (Raising Butterflies, Salt Lake City, UT, USA) and maintained at 26 °C, 60% relative humidity, and under a 16:8 h day/night photoperiod. Insects were supplied with water in a glass shell vial with a cotton wick and fed with green beans and tomatoes. Water was replenished as needed, and food was replaced thrice weekly. Adults used in this study were starved for 5 h prior to the experiments.

### 2.3. Experimental Design

In each potato plant, the terminal leaflet of the 4th leaf was used for the measurements [3]. The leaflet was enclosed in the measurement chamber of the fluorometer, and the chlorophyll fluorescence parameters were measured [3]. After the initial measurement, a single *H. halys* adult was placed on the leaflet within the measurement chamber, and a cap placed over it for enclosure [4,51]. After 20 or 90 min of feeding, the insect was removed, and the leaflet was measured immediately. In each treatment, 5 biological replicates (leaflets of different plants) were measured. In each leaflet, areas of interest (AOI) were selected before feeding, and new AOIs were added after feeding, with a total of 19–21 AOIs. ROS localization was evaluated before insect feeding, and immediately after the 3-, 5-, 10-, 20-, 30-, 60-, and 90-min feeding period.

### 2.4. Chlorophyll Fluorescence Imaging Analysis

In vivo chlorophyll fluorescence measurements were performed using an Imaging *PAM M-Series* system (*Heinz Walz GmbH*, Effeltrich, Germany), as described in detail before [4]. Dark-adapted (20 min) potato plants were measured before feeding (control), as well as 20 min and 90 min after feeding by *H. halys*. The minimum chlorophyll *a* fluorescence in the dark (F*o*) was measured with 0.5 μmol photons m^−2^ s^−^^1^ measuring light, and the maximum chlorophyll *a* fluorescence in the dark (F*m*) was obtained with a saturating pulse (SP) of 6000 μmol photons m^−2^ s^−1^ (470 nm, 800 ms) followed by application of blue LED (470 nm) actinic light (AL) of 640 μmol photons m^−^^2^ s^−^^1^ in order to match the growth light of potato plants. The chlorophyll fluorescence parameters that were calculated by the Imaging Win V2.41a software (*Heinz Walz GmbH*, Effeltrich, Germany) involved the effective quantum yield of PSII photochemistry (Φ*_PSII_*), the quantum yield of regulated non-photochemical energy loss (Φ*_NPQ_*), and the quantum yield of non-regulated energy (Φ*_NO_*), according to Krammer et al. [52], as described in detail before [3,4]. In addition, we measured the efficiency of excitation energy capture by open PSII centres (F*v*′/F*m*′) [53]; the redox state of quinone A (Q*_A_*), representing the fraction of open PSII reaction centres (q*p*) = (F*m*′ − F*s*)/(F*m*′ − F*o*′) [53]; the non-photochemical quenching (NPQ), reflecting the dissipation of excitation energy as heat, calculated as (F*m* − F*m*′)/F*m*′ [54]; the electron transport rate calculated as Φ_PSII_ × PAR × c × abs, where PAR is the photosynthetically active radiation, c is 0.5, and abs is the total light absorption of the leaf taken as 0.84 [55]; the excitation pressure (1−q*p*) [56]; and the excess excitation energy (EXC), calculated as (F*v*/F*m* − Φ_PSII_)/F*v*/F*m* [57].

Representative results such as colour-coded images of Φ*_PSII_*, Φ*_NPQ_*, Φ*_NO_*, and q*p* are also shown to reveal the whole leaf response to insect feeding and the spatial PSII heterogeneity before and after feeding.

### 2.5. Reactive Oxygen Species Imaging

We performed ROS detection in potato leaves before feeding and after 3-, 5-,10-, 20-, 30-, 60-, and 90-min of feeding, as described earlier [23]. In short, leaflets were excised and transferred to a 25 μΜ 2′,7′-dichlorofluorescein diacetate (DCF-DA, Sigma Aldrich, Chemie GmbH, Schnelldorf, Germany) aquatic solution followed by a 30 min incubation at about 25 °C in the dark for uptake in a rocking platform for equal fluorochrome distribution. When DCF-DA probe permeates membranes, it is oxidized by ROS, and the green fluorescence develops [23]. ROS production and distribution were visualized with a Zeiss AxioImager Z2 epi-fluorescence microscope equipped with an AxioCam MRc5 digital camera [3]. The relative DCF fluorescence was measured with excitation and emission wavelength set at 488 nm and 525 nm, respectively [3,23].

### 2.6. Statistics

Mean values were calculated for the 5 biological replicates of the two independent treatments (before and after feeding). The assumption of normality of data distribution was checked using the Shapiro–Wilk test, and the homogeneity of variance using Levene’s test, which showed unequal variances. Statistically significant differences among the means were determined using Welch’s ANOVA test. Means (±SD) were considered statistically different with *p* < 0.05 after a Game–Howell post-hoc test by using IBM SPSS Statistics for Windows version 28.

## 3. Results

### 3.1. The Light Energy Distribution at Photosystem II of Potato Leaf before and after Feeding

**Figure 1:**
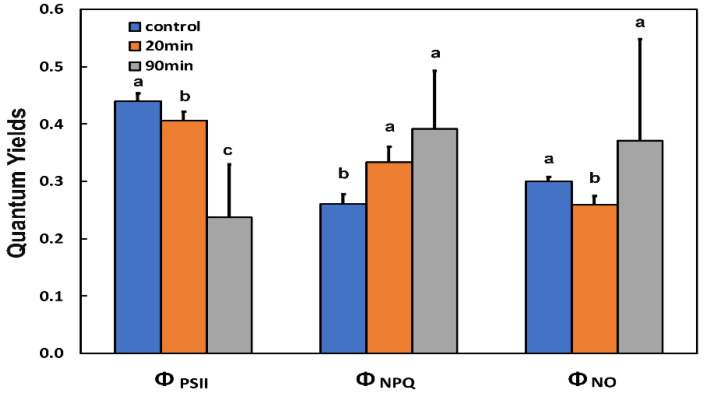
Changes in the allocation of absorbed light energy at photosystem II (Φ*_PSΙΙ_*, Φ*_NPQ_* and Φ*_NO_*) of potato leaves at the whole leaf level, before (control), and after 20 and 90 min feeding by the sap-sucking insect *Halyomorpha halys*. Error bars ± SD (*n* = 5). In each parameter, the columns with different lowercase letters are statistically different (*p* < 0.05).

After twenty min of feeding, the amount of energy that was used for photochemistry (Φ*_PSII_*) decreased significantly, while the 90 min feeding by *H. halys* decreased it further (Figure 1).

The allocation of absorbed light energy at PSII can be estimated by measuring the effective quantum yield of PSII photochemistry (Φ*_PSII_*), the quantum yield of regulated non-photochemical energy loss in PSII (Φ*_NPQ_*), and the quantum yield of non-regulated energy loss in PSII (Φ*_NO_*), the sum of all three to be equal to 1 [52]. The increased yield of regulated non-photochemical energy loss (Φ*_NPQ_*) at the whole leaf level after 20 min feeding was capable of overcompensating the decreased Φ*_PSII_* resulting in a decreased quantum yield of non-regulated energy (Φ*_NO_*) compared to before feeding (Figure 1), while after 90 min feeding, the increased Φ*_NPQ_* was sufficient enough to keep Φ*_NO_* at the same level as before feeding.

### 3.2. Changes in the Photoprotective Heat Dissipation, Electron Transport Rate, and the Redox State of the Plastoquinone Pool before and after Feeding

**Figure 2:**
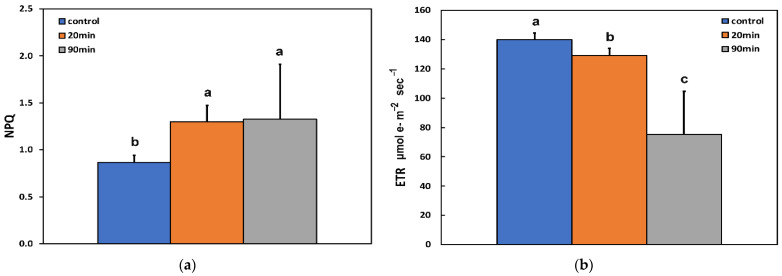
Changes in (**a**) NPQ; and (**b**) ETR (in μmol electrons m^−2^ s^−1^); of potato leaves, at the whole leaf level, before (control) and after 20 and 90 min feeding by *Halyomorpha halys*. Error bars ± SD (*n* = 5). In each parameter, the columns with different lowercase letters are statistically different (*p* < 0.05).

Non-photochemical quenching (NPQ), which reflects the dissipation of excitation energy as heat, increased after 20 min feeding compared to before feeding, but did not increase further at longer durations of feeding (90 min) (Figure 2a). The electron transport rate (ETR) decreased with increasing feeding time compared to before feeding (Figure 2b).

**Figure 3:**
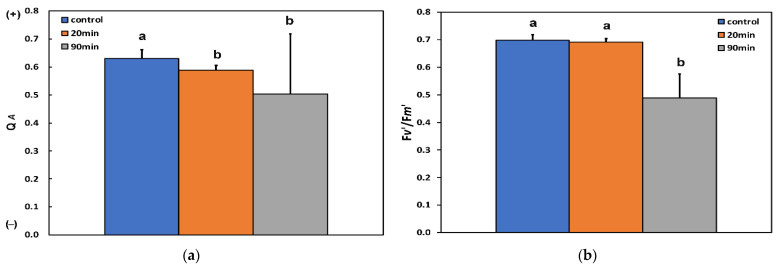
Changes in (**a**) the Q*_A_*; and (**b**) the F*v*′/F*m*′; of potato leaves, at the whole leaf level, before (control) and after 20 and 90 min feeding by *Halyomorpha halys*. Error bars ± SD (*n* = 5). In each parameter, the columns with different lowercase letters are statistically different (*p* < 0.05).

The redox state of quinone A (Q*_A_*) at the whole leaf level, representing the fraction of open PSII reaction centres (q*p*), decreased after 20 min feeding compared to before feeding, but remained the same at longer durations of feeding (90 min) (Figure 3a).

The efficiency of excitation energy capture by open PSII centres (F*v*′/F*m*′) at the whole leaf level after 20 min feeding remained the same as before feeding but decreased after 90 min feeding (Figure 3b).

### 3.3. Changes in the Efficiency of Open Photosystem II Reaction Centers, the Excitation Pressure, and the Excess Excitation Energy in Photosystem II before and after Feeding

**Figure 4:**
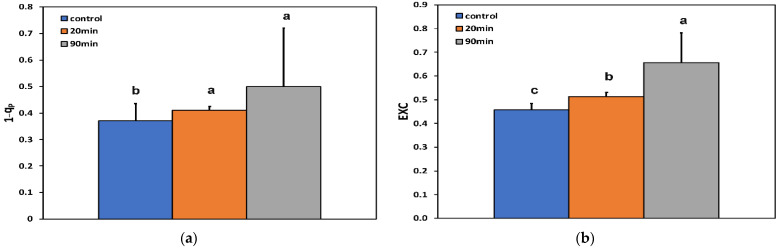
Changes in (**a**) the 1−q*p* (data of Figure 3a) and (**b**) the EXC of potato leaves, at the whole leaf level, before (control) and after 20 and 90 min feeding by *Halyomorpha halys*. Error bars ± SD (*n* = 5). In each parameter, the columns with different lowercase letters are statistically different (*p* < 0.05).

The excitation pressure at PSII (1−q*p*) at the whole leaf level increased after 20 min feeding compared to before feeding, but did not increase further at the longer duration of feeding (90 min) (Figure 4a).

The excess excitation energy (EXC), calculated as (F*v*/F*m* − Φ*_PSII_*)/F*v*/F*m* [52], increased with increasing feeding time compared to before feeding (Figure 4b).

### 3.4. The Spatial Pattern of Photosystem II Activity of Potato before and after Feeding

**Figure 5:**
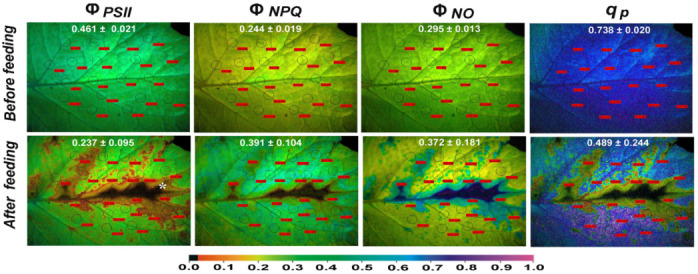
Typical colour-coded images of Φ*_PSΙΙ_*, Φ*_NPQ_*, Φ*_NO_*, and q*p*, of a potato leaflet before insect feeding (**upper row**) and immediately after 90 min feeding (**second row**) by *Halyomorpha halys*. The areas of interest (AOIs) that were measured are shown in circles with values in red labels. The whole leaflet values (average ± SD) are given with the white numbers. In the image of Φ*_PSΙΙ_* after 90 min feeding; asterisk indicates Φ*_PSII_* value 0.064. The colour code at the bottom ranges from pixel values 0.0 to 1.0.

After 90 min feeding, an increased spatial PSII heterogeneity was observed in potato leaves (Figure 5). Φ*_PSII_* decreased significantly at the feeding zones that were located almost exclusively at the main vein and especially at the sieve elements and the neighbouring area (Figure 5) with the feeding spot.

The area that was detrimentally affected by the insect feeding (marked by an asterisk in Figure 5) had a Φ*_PSII_* value of 0.064. Yet, Φ*_PSII_* decreased at the whole leaf level (Figure 1, Figure 5, and Figure S2). At the same time, Φ*_NPQ_* increased significantly at the whole leaf level (Figure 1, Figure 5, and Figure S3), but decreased significantly at the feeding zones compared to before feeding (Figure 5 and Figure S3).

After 90 min feeding, the decreased Φ*_PSII_* and Φ*_NPQ_* at the feeding zones resulted in a significant increase of Φ*_NO_* at these zones, especially at the main vein (Figure 5 and Figure S4). In the neighbouring area to the feeding zone, Φ*_NPQ_* increased more than in the rest of the leaf area to compensate for the higher decrease of Φ*_PSII_* in the same zone (Figure 5, Figure S2, and Figure S3). Still, at the whole leaf level, due to the significant increase of Φ*_NPQ_*, there was no significant change in Φ*_NO_* (Figure 1). The number of open PSII reaction centres decreased significantly at the whole leaf level after feeding (Figure 1 and Figure 5), with the decrease being more intense in the feeding zones (Figure 5). However, a little far from the feeding area, there was a significant increase compared to before feeding in the number of open reaction centres of PSII (q*p*) (Figure 5 and Figure S5).

Twenty minutes after feeding, a decreased Φ*_PSII_* was also noticed (Figure 1), but with much less spatial heterogeneity compared to the 90 min feeding (Figures S2 and S6). The same was also true for the chlorophyll fluorescence parameters Φ*_NPQ_* and Φ*_NO_* compared to the 90 min feeding (Figure S3, Figure S4, and Figure S6).

### 3.5. Reactive Oxygen Species Localisation before and after Feeding

**Figure 6:**
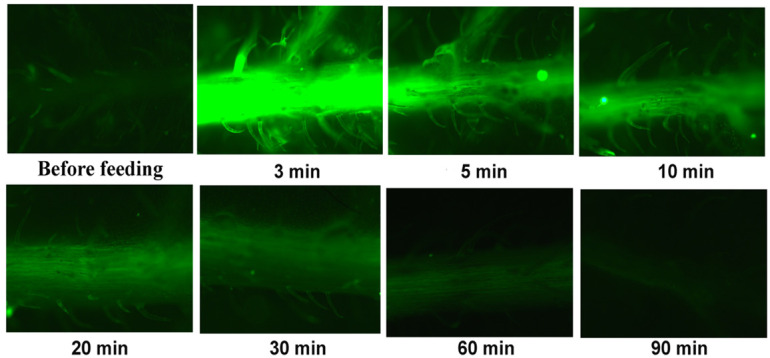
Reactive oxygen species (ROS) localization by staining of potato leaflet with 2′,7′-dichlorofluorescein diacetate (DCF-DA) before and after 3-, 5-,10-, 20-, 30-, 60-, and 90-min of feeding by *Halyomorpha halys*. Increased ROS generation visible by light green colour.

ROS imaging, before feeding and 3-, 5-,10-, 20-, 30-, 60-, and 90-min after feeding, performed by staining the potato leaves with 25 µM 2′,7′-dichlorofluorescein diacetate in the dark, revealed an intense increase of ROS generation that was visible as green fluorescence as soon as 3 min after feeding (Figure 6). Reactive oxygen species before feeding were vaguely detected in leaf trichomes. Meanwhile, with the 3 min feeding, an increased generation was detected almost exclusively in the main leaf veins and the leaf trichomes (Figure 6). The ROS real-time staining pattern revealed a decreased generation with further elapsed time feeding, while after 90 min feeding time, ROS visualization was undetectable (Figure 6).

## 4. Discussion

Herbivory is one of the most important factors affecting plant fitness [58], and plant primary photochemistry is the principal plant trait shaping plant–herbivore interaction. The response mechanism of photosystem II photochemistry to insect herbivory and its ability to induce a compensatory mechanism that increases PSII photochemistry in response to herbivore feeding is fundamental in plant resistance to herbivores [4]. The light reactions of photosynthesis involve a set of redox reactions that are the source of energy for producing organic compounds [59]. According to the growth-differentiation hypothesis, plants have to choose between allocating their resources to defence or growth [60]; greater allocation of resources to defence comes at the expense of tolerance [61]. Induction of plant response to herbivores is explained by two traits, resistance and tolerance. Induction of resistance results in reduced herbivore damage, while induction of tolerance through the increase in growth and photosynthesis compensates for herbivore damage, reducing the negative fitness impact of injury [8,62].

The decrease in the quantum yield of PSII photochemistry (Φ*_PSII_*) that was observed after 20 min feeding (Figure 1), according to Genty et al. [53], can be attributed either to a decreased fraction of open PSII reaction centres (q*p*) (a measure of the redox state of quinone A (Q*_A_*)) or to a decrease in the efficiency of these centres (F*v*′/F*m*′) (the supply of energy reaching the PSII reaction centres) [53,63]. In our case, considering that there was no significant change in the efficiency of open PSII reaction centres after 20 min feeding (Figure 3b), it is concluded that the decreased Φ*_PSII_* was due to a decreased fraction of open PSII reaction centres (Figure 3a). However, after 90 min feeding, the decreased Φ*_PSII_* was due to both a decreased efficiency of the open PSII reaction centres (F*v*′/F*m*′) (Figure 3b) and a more reduced state of Q*_A_* (Figure 3a) compared to before feeding. Non-photochemical quenching (NPQ) mechanism can reduce the energy transfer to reaction centres, thus reducing the efficiency of PSII centres [63]. The NPQ parameter represents mainly heat dissipation from the light-harvesting complex II (LHCII) via the zeaxanthin quencher [64,65]. This heat dissipation decreases the efficiency of photochemical reactions of photosynthesis (down-regulation of PSII) [22,23,66,67].

The increased NPQ, 90 min after feeding, decreased Φ*_PSII_* due to reduced efficiency of PSII centres (F*v*′/F*m*′) and decreased the electron-transport rate (ETR) in order to prevent ROS formation [68]. The increased ROS generated as soon as 3 min after feeding (Figure 6) was shown to diffuse through the leaf veins to act as a long-distance signalling molecule [23,69,70,71]. ROS signalling pathways are induced by the redox state of Q*_A_*, also comprising a mechanism of plant acclimation by regulating photosynthetic gene expression [23,72,73,74]. The redox state of Q*_A_* is of rare significance for antioxidant defence and signalling [75]. For example, the reduced state of Q*_A_* is suggested to mediate stomatal closure, probably by ROS generation, conferring acclimation to Cd exposure [76,77]. Reactive oxygen species (ROS) are now recognized as signalling molecules, with an essential part in numerous cellular processes having a tight control exerted by the antioxidant machinery and triggering signalling mechanisms governing normal growth and development and in response to external abiotic or biotic stresses [40,63,78,79,80,81].

The amount of absorbed light energy used in photochemistry (Φ*_PSII_*) after 20 min feeding, at the whole leaf level, was lower by 8% compared to before feeding (Figure 1) and by 46% after 90 min feeding. The decrease in Φ*_PSII_* after 20 min feeding was overcompensated by the increase in the photoprotective energy dissipation (Φ*_NPQ_*) that resulted in a lower Φ*_NO_* (Figure 1). The non-regulated energy loss in PSII (Φ*_NO_*) encompasses the energy of the triplet-state chlorophylls (^3^Chl*), which is generated through the intersystem crossing of the singlet excited chlorophyll state (^1^Chl*) and is transferred to molecular oxygen (O_2_), to generate singlet-excited oxygen (^1^O_2_) [79,82,83,84,85,86,87]. Reactive oxygen species, estimated as ^1^O_2_, after 20 min feeding decreased (Figure 1), but total ROS generation, as observed with 2′,7′-dichlorofluorescein diacetate (DCF-DA) staining, increased compared to before feeding (Figure 6). As ROS formed either by energy transfer (^1^O_2_) and/or electron transport (O_2_^•−^, H_2_O_2_) are created concurrently, it appears likely that their action of signalling pathways can occasionally interfere or antagonize each other [23,40,45,74,79,83].

After 90 min feeding by the sap-sucking insect *H. halys*, Φ*_PSII_* at the whole leaf level decreased by 46%. Meanwhile, at the feeding zones, which were located in the proximity of the leaf’s midrib, Φ*_PSII_* at the spot-like area of the feeding zone was lower by 85%, as judged by the Φ*_PSII_* value of 0.064 (Figure 5). However, despite a significant increase of Φ*_NPQ_* at the whole leaf level (Figure 1 and Figure 5), at the feeding zones, it decreased significantly compared to before feeding (Figure 5). Consequently, the decreased Φ*_PSII_* and Φ*_NPQ_* at the feeding zones resulted in a significant increase of Φ*_NO_* at these zones (Figure 5), indicating high ^1^O_2_ generation, mostly at the main vein and the neighbouring area (Figure 5). Singlet-excited oxygen (^1^O_2_) is considered a harmful ROS produced by PSII that inhibits the repair of PSII reaction centres and/or contributes directly to PSII damage [70,71,79], while increased ^1^O_2_ generation can trigger programmed cell death [3,88]. Singlet-excited oxygen (^1^O_2_) is a powerful oxidant that reacts rapidly in the area where it is created, resulting in oxidation that is regularly denoted as “damage” [20]. When the rate of damage exceeds the rate of D1 protein repair, the consequence is an overall decrease in ETR [69]. In our case, after a 90 min feeding, the destructive role of ROS was due to ^1^O_2_ generation (increased Φ*_NO_*) predominantly at the main vein area (Figure 5).

The highly increased ROS generation at the feeding zones located in the proximity of the leaf’s midrib, as soon as 3 min after feeding (Figure 6), triggers defence responses through leaf vein diffusion since ROS (and especially H_2_O_2_) act as long-distance signalling molecules [23,70,71,73]. Hydrogen peroxide is the most stable ROS that can mediate plant responses to stress [89]. A controlled ROS burst has been considered the primary plant defence responses mechanism against herbivores [90]. A quick activation of plant defence signals in the cells surrounding the stylet wound by an aphid in *Arabidopsis thaliana* was followed by an induction spread along the veins to the whole leaf [91]. In the neighbouring area to the feeding zone, 90 min after feeding, the photoprotective mechanism Φ*_NPQ_* increased more than in the rest of the leaf area to compensate for the higher decrease of Φ*_PSII_* in the same zone. At the whole leaf level, due to the significant increase of Φ*_NPQ_* after 90 min feeding, there was no significant change of Φ*_NO_*, which represents ^1^O_2_ generation, which remains the same as before feeding.

Reactive oxygen species generation can inhibit the repair of PSII reaction centres or contribute directly to PSII damage [42,63]. ROSs act primarily by inhibiting the repair of photodamaged PSII and not by damaging PSII directly [92]. However, ROS production in chloroplasts not only generates oxidative stress, but also presents essential biological functions in plant growth, development, and redox signalling [40,93,94]. A proper equilibrium between the creation and removal of ROS by chloroplast antioxidants is achieved so as to match the process of photosynthesis, allowing an effective scattering of ROS signals [3,45,79,95]. Consequently, ROS not only compromises cells with tools to monitor electron transport and, thus, avoid over-reduction or over-oxidation, but also creates redox regulatory networks that enable plants to sense and react to biotic and abiotic stress conditions [39,70,79,95,96]. A high level of ROS is considered to be useful for initiating defense responses [45,80,95]. Photosystem II responses under biotic or abiotic stress are activated by the NPQ mechanism that is a strategy to protect the chloroplasts from photo-oxidative damage by heat dissipation [80,97,98], regulating ETR [71,95], and avoiding detrimental ROS generation [66,80]. A basal level of ROS is required for optimum plant growth [40,45], with a controlled, increased ROS level to be favourable in activating defence responses, while a high level of ROS out of the limits to be destructive to plants [3,40,45,80].

Our experimental results confirmed that chlorophyll fluorescence imaging methodology can detect spatial heterogeneity of PSII efficiency at the whole leaf surface and is a promising tool for investigating plant response mechanisms to sap-sucking insect herbivores. As it has been previously suggested, it can distinguish leaf photosynthetic spatiotemporal heterogeneity that cannot be identified through conventional chlorophyll fluorescence analysis, and it can be used to explore plant response mechanisms to biotic or abiotic stresses [50,80,99,100,101,102,103]. Plant-insect interactions are getting more defined and comprehensive with the development of novel visualization methods that permit leaf photosynthetic efficiency evaluation after herbivore attack [4,91].

## 5. Conclusions

We suggest that PSII responses after feeding by *H. halys* underlie ROS-dependent signalling that occurs immediately (<3 min) after feeding. It is concluded that the potato PSII response mechanism to sap-sucking insect herbivores is a defence response that reduces damage to herbivory instead of the induction of tolerance through a compensatory photosynthetic response mechanism that was observed in chewing insects [4,51].

## Data Availability

The data presented in this study are available in this article.

## Supplementary Materials

The following supporting information can be downloaded at: https://www.mdpi.com/article/10.3390/insects13050409/s1. Figure S1: Dorsal view of the brown marmorated stink bug (Halyomorpha halys). Figure S2: Histogram of all data points of the effective quantum yield of PSII photochemistry (ΦPSII) per time point. Figure S3: Histogram of all data points of the quantum yield of regulated non-photochemical energy loss (ΦNPQ) per time point. Figure S4: Histogram of all data points of the quantum yield of non-regulated energy (ΦNO) per time point. Figure S5: Histogram of all data points of the fraction of open PSII reaction centres (qp) per time point. Figure S6: Typical colour-coded images of Φ*_PSΙΙ_*, Φ*_NPQ_*, and Φ*_NO_*, before insect feeding and immediately after 20 min feeding.
