# Supplementary material for: Reactive Oxygen Species Initiate Defence Responses of Potato Photosystem II to Sap-Sucking Insect Feeding
Source: Insects. 2022 Apr 24;13(5):409. doi: 10.3390/insects13050409 (PMC9147889; doi:10.3390/insects13050409)
Supplement: Supplementary file 1 [file insects-13-00409-s001.zip › insects-1687856-SI.pdf]

Reactive Oxygen Species Initiate Defence Responses of Potato Photosystem II to Sap-Sucking Insect Feeding

Ilektra Sperdouli [1], Stefanos Andreadis [1], Ioannis-Dimosthenis S. Adamakis [2,*], Julietta Moustaka [3,4], Eleni Koutsogeorgiou [1], and Michael Moustakas [3,*]

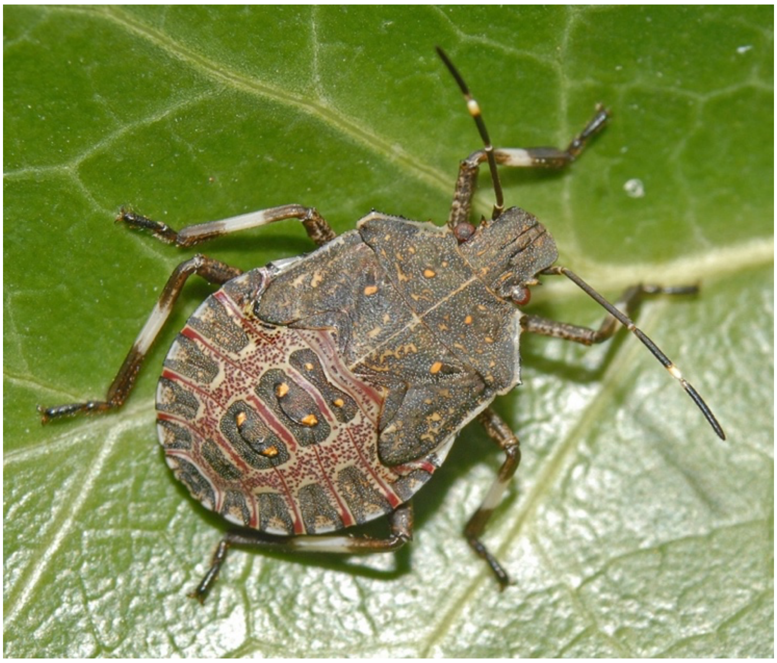

Figure S1. Dorsal view of fifth instar nymph of the brown marmorated stink bug (*Halyomorpha halys* Stål) an insect of the Pentatomidae family, feeding at the main vein (https://antropocene.it/en/2019/11/01/halyomorpha-halys/)

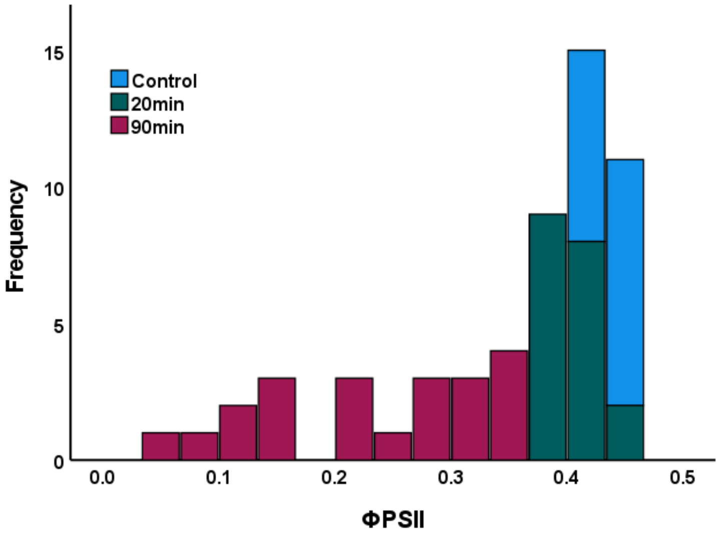

Figure S2. Histogram of all data points of the effective quantum yield of PSII photochemistry (Φ_{PSII}) per time point (data of Figure 1).

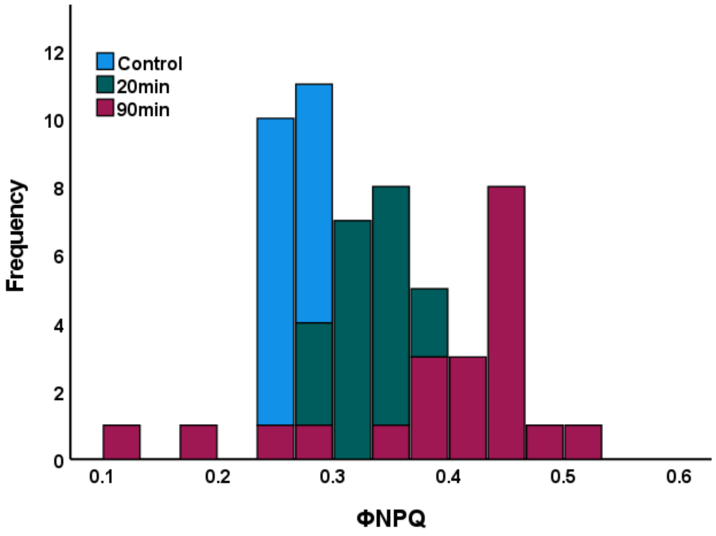

Figure S3. Histogram of all data points of the quantum yield of regulated non-photochemical energy loss (Φ_{NPQ}) per time point (data of Figure 1).

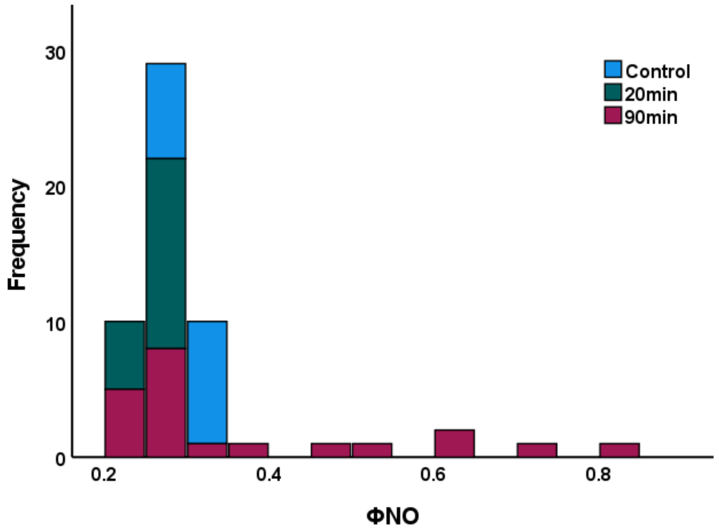

Figure S4. Histogram of all data points of the quantum yield of non-regulated energy (Φ_{NO}) per time point (data of Figure 1).

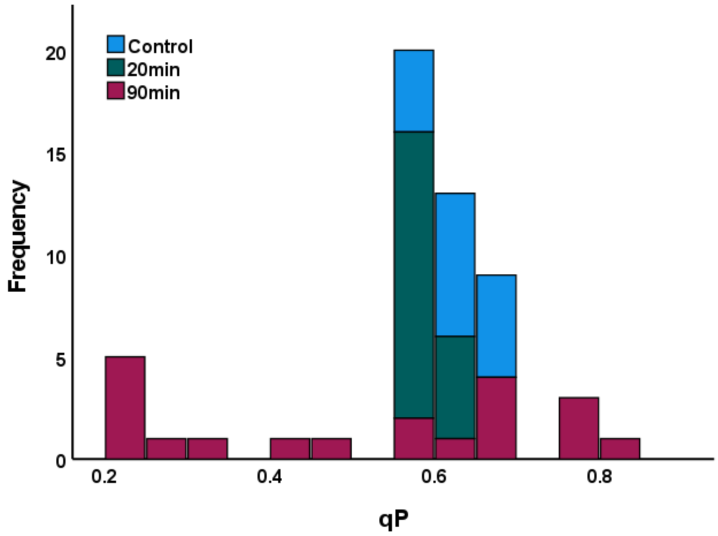

Figure S5. Histogram of all data points of the fraction of open PSII reaction centers (q*p*) per time point (data of Figure 3a).

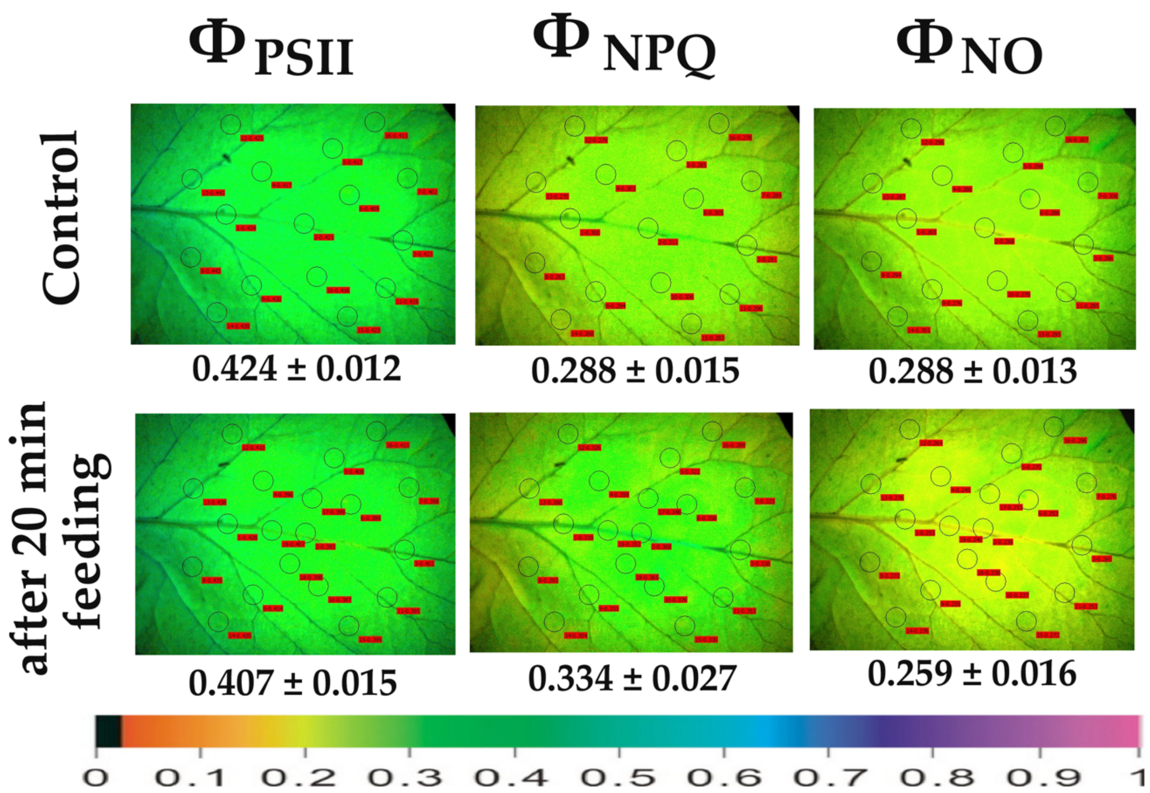

Figure S6. Typical color-coded images of the effective quantum yield of PSII photochemistry (Φ_{PSII}), the quantum yield of regulated non-photochemical energy loss (Φ_{NPQ}), and the quantum yield of non-regulated energy loss (Φ_{NO}), before insect feeding (control), and immediately after 20 min feeding by the sap-sucking insect *Halyomorpha halys*. The areas of interests (AOIs) are shown in circles with their associated measurements in red labels; the resultant values for the whole leaflet (average ± SD) are given also. The color code at the bottom ranges from pixel values 0.0 to 1.0.
